# Clinical study of modified INFIX combined with sacroiliac joint screws for pelvic instable injuries

**DOI:** 10.1186/s12893-023-02205-1

**Published:** 2023-11-16

**Authors:** Peishuai Zhao, Renjie Li, Leyu Liu, Xiaopan Wang, Xiaotian Chen, Jianzhong Guan, Min Wu

**Affiliations:** Department of Orthopaedics, The First Affiliated Hospital of Bengbu Medical College, Bengbu, Anhui China

**Keywords:** Pelvic fracture, Internal fixation, Minimal invasive surgery, Complications, Lateral femoral cutaneous nerve

## Abstract

**Objective:**

The INFIX technique is becoming one of the most commonly performed surgical procedures for anterior pelvic ring instability injuries. The purpose of this article is to compare the clinical outcomes of modified anterior subcutaneous internal fixation (M-INFIX) with conventional anterior subcutaneous internal fixation (C-INFIX) for anterior pelvic ring instability injuries.

**Patients and methods:**

A retrospective analysis of 36 cases of unstable pelvic injuries treated operatively at our institution, 20 of which were treated with C-INFIX and 16 with M-INFIX. Data collected included age, gender, ISS score, fracture typing, operative time, operative bleeding, postoperative complications, fracture healing time, Matta score, Majeed score, and follow-up time. Statistical sub-folding of each variable between the two groups was performed.

**Results:**

There was no statistical difference between the C-INFIX and M-INFIX groups in terms of age, gender, ISS (Injury Severity Score), follow-up time, fracture typing, fracture healing time, and Majeed score (P > 0.05). the M-INFIX had a significantly lower incidence of postoperative complications than the C-INFIX group, especially in the incidence of Lateral femoral cutaneous nerve (LFCN) injury (P < 0.05). In contrast, the M-INFIX group had statistically higher operative time, intraoperative bleeding, and Matta score than the C-INFIX group (P < 0.05).

**Conclusion:**

This study was based on a modified application of the surgical experience with C-INFIX and showed better clinical outcomes in terms of complication rates and quality of repositioning than the conventional surgical approach. These findings indicate that further analytical studies of this study would be valuable.

## Introduction

Pelvic fractures account for approximately 0.3-6% of all fractures, 20% of which are multiple injuries [1]. Most of the anterior pelvic rings are located subcutaneously and are easily damaged when subjected to external forces, resulting in a breakdown of the integrity and stability of the pelvis [2, 3]. Currently, the mainstream treatment methods for anterior pelvic ring injuries include external fixation, internal fixation with incisional repositioned plates, and INFIX [4]. External pelvic fixation frame is commonly used in patients with multiple trauma and hemodynamic instability, which can quickly stabilize and reduce the volume of the pelvic ring, reduce the “chimney effect” caused by pelvic ring rupture, and reduce bleeding [5]. However, the stability of the posterior pelvic ring is poorly controlled by external pelvic fixation, and the posterior ring still needs to be fixed at a later stage [1]. Moreover, the external fixation frame protrudes from the skin surface, which affects the patient’s turning activities and daily life, and there may be adverse complications such as loosening of the nail path and infection in the future [6]. According to several reports, the complication rate of external fixation frame is between 12% and 58% [7]. Therefore, external pelvic fixators are often not used clinically as the final fixation for pelvic ring injuries [8].

Many scholars believe that incision and plate fixation of the anterior pelvic ring is the best treatment method because the fracture end can be repositioned under direct vision after incision, and the plate and screws can obtain good repositioning effect and biomechanical stability [4, 9]. However, the disadvantages of large bleeding, vascular nerve injury, incisional infection, extensive periosteal debridement, and complicated surgical operation have seriously limited the further application of this technique [10].

In this background, Kuttner et al. first reported the use of an anterior subcutaneous internal fixator (INFIX) to try to address these problems, which was invented using a similar fixation principle to that of a conventional external fixator, but is placed entirely subcutaneously [11]. Biomechanical studies have shown that the INFIX has similar or greater stability than an external fixator, and it also has a lower infection rate and is easier to care for [6]. In recent years, as technology has advanced, the indications have expanded [12, 13]. Unfortunately, INFIX is not without complications. Lateral femoral cutaneous nerve palsy (LFCN), femoral nerve palsy, loosening of the implant, and heterotopic ossification are some of the problems that can occur after this procedure [14].

**Fig. 1:**
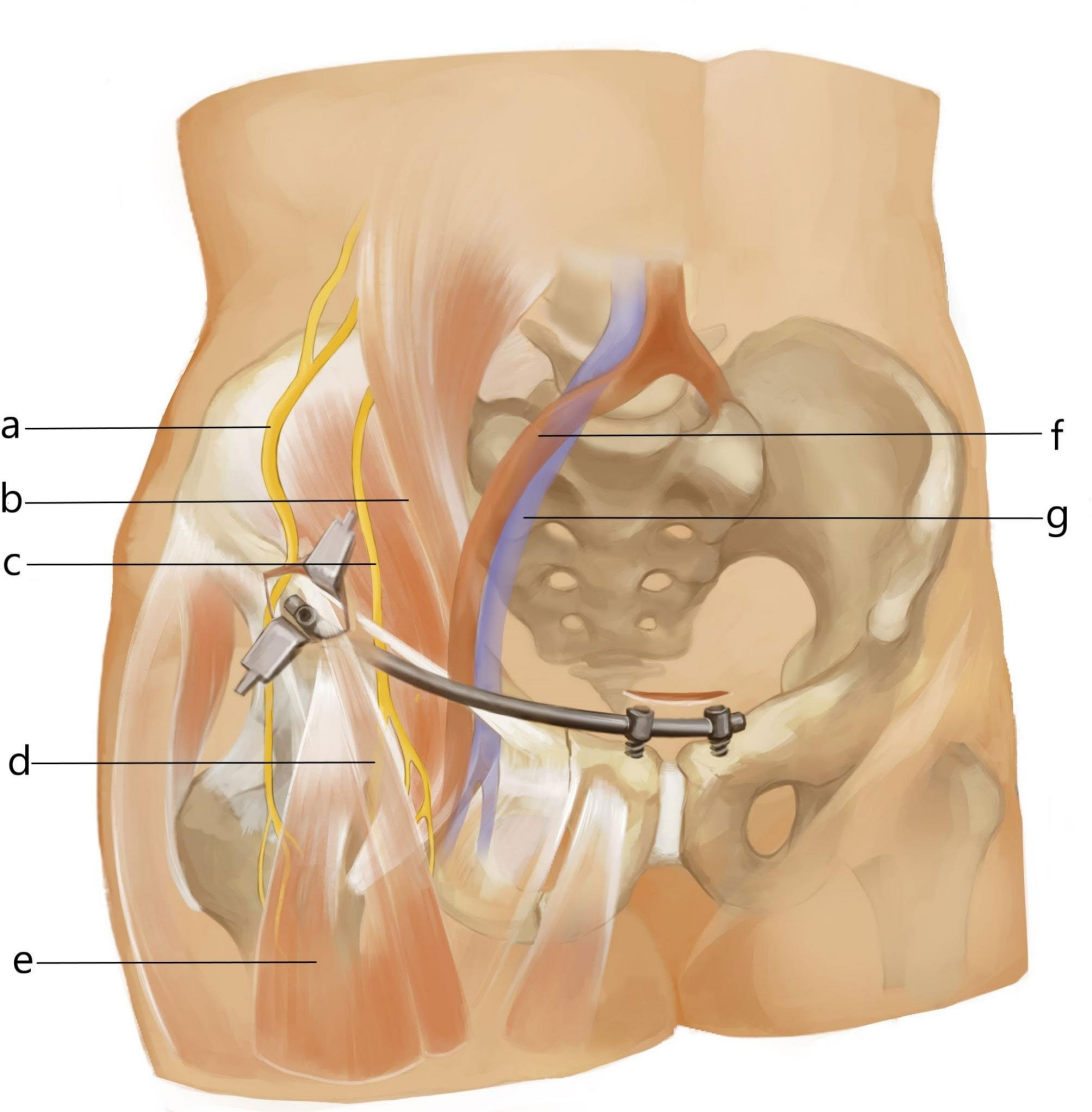
The incision designed for M-INFIX. The two incisions are located on the anterior inferior iliac spine and over the pubic symphysis on one side, and the multiaxial screws are placed medially in the anterior inferior iliac spine and bilaterally in the pubic symphysis. A subcutaneous tunnel is created from the anterior inferior iliac spine incision toward the pubic symphysis, which allows manual insertion of titanium rods. (**a**) Lateral femoral cutaneous nerve; (**b**) Iliopsoas muscle; (**c**) Femoral nerve; (**d**) suture muscle; (**e**) Quadriceps muscle; (**f**) External iliac artery; (**g**) External iliac vein

In previous studies, the incidence of these complications was found to range from 0–40% [15]. Despite a series of anatomical studies on INFIX, there is no clear procedural maneuver that can effectively reduce the rate of complications in previous reports [16]. Therefore, we propose a new method to fix the anterior pelvic ring using a modified INFIX pedicle screw system from the ilium up to the pubic symphysis bilaterally (Fig. 1). To the best of our knowledge, no one has compared the complication rates and related clinical outcomes of C-INFIX and M-INFIX. The purpose of this study was to compare the short-term results of two different protocols for the treatment of anterior pelvic ring instability injuries.

## Patients and methods

This study was approved by the ethical institutional committee (Number: BYYFY-2022KY306). Thirty-six patients with unilateral pelvic instability injuries treated at our institution from February 2018- February 2022 received C-INFIX or M-INFIX. Inclusion criteria: (1) unilateral posterior pelvic ring instability injury combined with unilateral anterior ring injury, type AO/OTA61B1, B2, C1, posterior pelvic ring fixed with sacroiliac joint screws and ensuring that the posterior pelvic ring has adequate stability; (2) age > 16 years; (3) follow-up time > 6 months; exclusion criteria: (1) bilateral anterior pelvic ring injury; (2) age < 16 years; (3) combined acetabular fracture; (4) infection in the surgical area; (5) open pelvic fracture.

Preoperatively, pelvic anteroposterior, inlet, and outlet radiographs, computerized thin-section computed tomography (CT) scans, and fracture typing according to the OTA/AO classification were all routinely examined by two experienced trauma orthopedic surgeons.

### Surgical method

**Fig. 2:**
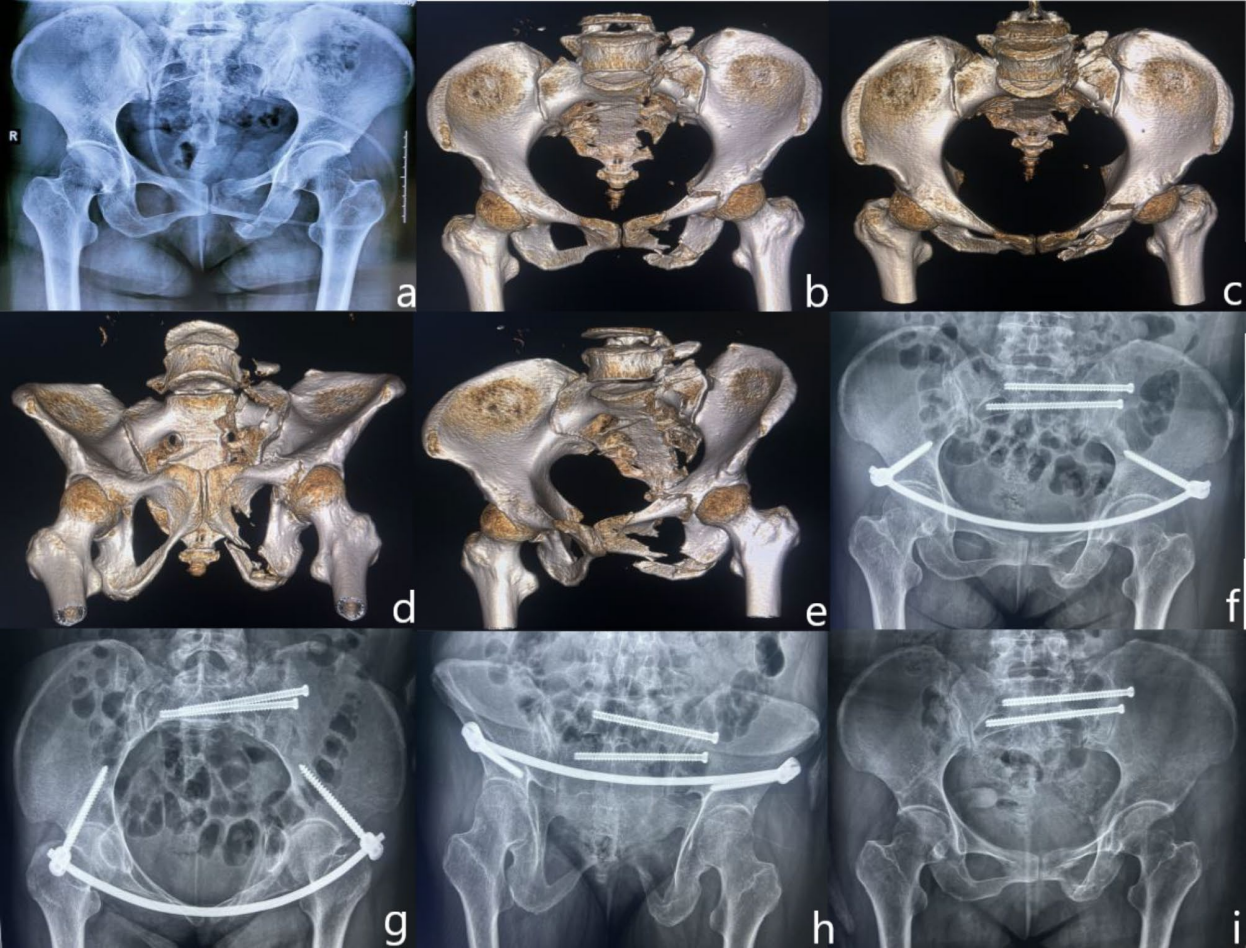
C-INFIX patient, female, 41 years old, preoperative diagnosis: OTA/AO 61B1.3. Posterior pelvic ring fixed by sacroiliac joint screws, anterior ring fixed with C-INFIX, titanium rod shaped according to Bikini area. (**a**) Preoperative anterior-posterior pelvic radiographs; (**b**) Preoperative 3D reconstructed anterior-posterior pelvic views; (**c**) 3D reconstructed pelvic inlet views; (**d**) 3D reconstructed pelvic outlet views; (**e**) Preoperative 3D reconstructed closed-hole oblique views; (**f**) Postoperative anterior-posterior pelvic radiographs; (**g**) Postoperative pelvic inlet radiographs; (**h**) Postoperative pelvic outlet radiographs; (**i**) Postoperative anterior-posterior pelvic radiographs after removal of the internal fixation

A variable number of sacroiliac screws are placed in all cases involving posterior pelvic ring instability injuries, depending on the case. The C-INFIX surgical technique has been described in detail by Vaidya and is used clinically to date [17]. A 2–3 cm long oblique incision was made centered on one anterior inferior iliac spine, and the muscle space between the tensor fascia latae and sartorius was bluntly separated to expose the anterior inferior iliac spine and its underside. Entry was made with an open cone approximately 3 cm below the inner anterior inferior iliac spine, oriented at a 20–30 degree caudal and medial inclination, and a probe was used to enter between the inner and outer iliac bony plates. Fluoroscopy of the outlet obturator oblique and outlet iliac oblique radiographs ensured that the probe was in the correct position, and one multiaxial pedicle screw was screwed in, and the contralateral multiaxial pedicle screw was screwed in the same way. Both hands were placed at the anterior superior iliac spine on both sides of the patient to perform manipulation and repositioning. After the fracture was satisfactorily repositioned under fluoroscopy, a titanium rod of appropriate length was shaped to the curvature of the patient’s lower abdomen in the Bikini region, inserted into the titanium rod along the deep fascial surface of the skin, and attached to the pedicle screws bilaterally after the repositioning was maintained. The screws of the healthy pelvis were locked as a priority during fixation, and the screws of the injured pelvis were locked when intraoperative fluoroscopic observation of the fracture break was well aligned and aligned. Make sure that the pedicle screws are 2 cm away from the bone surface and that the skin can be sutured loosely to reduce the possibility of local skin necrosis (Fig. 2).

**Fig. 3:**
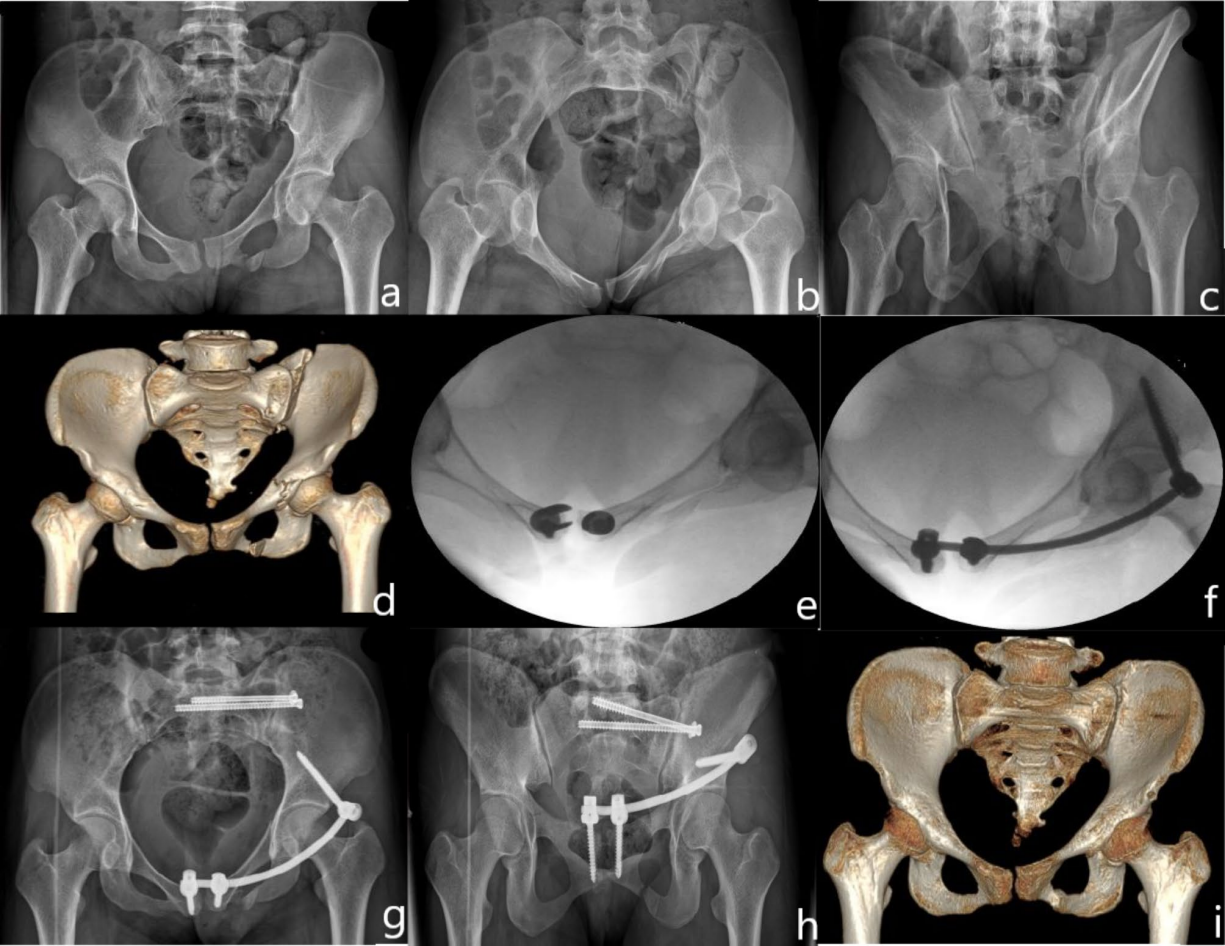
Patient in the M-INFIX group, female, 28 years old, preoperative diagnosis: OTA/AO 61C1.3. The posterior pelvic ring was fixed with 2 sacroiliac screws and the anterior ring was fixed with M-INFIX, shaped according to the Bikini area of the hemi-pelvis. (**a**) Preoperative anterior-posterior pelvic radiograph; (**b**) Preoperative pelvic inlet radiograph; (**c**) Preoperative pelvic outlet radiograph; (**d**) Preoperative anterior-posterior 3D reconstruction of the pelvis; (**e**) Intraoperative placement of 2 multiaxial screws on both sides of the pubic symphysis; (**f**) Intraoperative inlet radiograph; (**g**) Postoperative anterior-posterior pelvic radiograph; (**h**) Postoperative pelvic outlet radiograph; (**i**) Postoperative 3D reconstruction after removal of the M-INFIX at 4 months, showing that the fracture had healed

The M-INFIX is a moderate modification of the C-INFIX, which consists of three pedicle screws and a titanium rod. A 2–3 cm long oblique incision was made centered on the anterior inferior iliac spine of the injured hemipelvis, and the muscle space between the tensor fasciae latae and the sartorius was bluntly separated. Expose the anterior inferior iliac spine and its underside, and use the aperture device to open the anterior inferior iliac spine approximately 3 cm inferiorly from the inside, oriented at a 20–30 degree caudal and medial inclination. Fluoroscopy of the outlet obturator oblique and outlet iliac oblique radiographs ensured that the probe was in the correct position, and one multiaxial pedicle screw was screwed in; An incision of approximately 4–5 cm in length is made above the pubic symphysis and bluntly separated until both pubic tubercle are exposed. The entry point is located 1 cm lateral to the pubic tubercle, oriented with a 25–35 degree inclination toward the anterior side, and the probe is used to enter between the internal and external pubic plates. Fluoroscopic pelvic inlet and outlet radiographs were taken to ensure that the probes were in the correct position, and 2 pedicle screws were placed into the osseous channel respectively (Fig. 1). The operator placed one hand at the anterior superior iliac spine on the injured side and the other hand at the pubic symphysis screw on the injured side to perform manipulative repositioning. After the fracture was satisfactorily repositioned by fluoroscopy, the titanium rods were inserted along the deep fascial surface of the skin according to the curvature of the Bikini region of the patient’s hemi-abdomen, and the titanium rods were attached to the 3 pedicle screws after maintaining the repositioning. The lateral iliac screws are first locked and the 2 screws next to the pubic symphysis are sequentially locked after good intraoperative fluoroscopic repositioning of the fracture end. The lateral iliac screws should be kept at least 2 cm from the bone surface, but the 2 screws at the pubic symphysis should be screwed to a depth determined by the patient’s lower abdominal skin augmentation (Fig. 3).

### Postoperative management

Patients were routinely given antibiotics within 24 h to prevent infection, encouraged to perform functional exercises of the quadriceps muscle in bed within 3 days and given anti-thrombotic drugs, and the surgical sutures were removed in about 2 weeks. The pelvis should be reviewed regularly at 1 month, 2 months, 3 months, 6 months, and 1 year after surgery with anterior-posterior, closed-hole exit, and entry-exit radiographs, and CT scan of the pelvis as necessary. It is recommended to remove the internal fixation after fracture healing at 3 months postoperatively. The degree of repositioning was assessed by postoperative radiographs according to Matta criteria (excellent: displacement ≤ 4 mm, good: displacement 5–10 mm, fair: 10–20 mm, poor: displacement > 20 mm [18]. Functional outcome at the final follow-up was assessed using the Majeed score (pain: 30 points, standing: 36 points, sitting: 10 points, intercourse: 4 points and work: 20 points). The final score was divided into 4 stages, excellent (> 85), good (70–84), fair (55–69) and poor (< 55) [19].

### Statistical Data Analysis

The main outcome indicators were the occurrence of postoperative adverse complications and radiological findings. Patients’ gender, age, ISS, fracture typing, operative time (from the beginning to the end of anterior pelvic ring fixation), intraoperative bleeding, complications, follow-up time, fracture healing time, Matta score, and Majeed score were also recorded. Data were analyzed using SPSS version 22.0 software (SPSS, Chicago, IL, USA). Data satisfying the normality condition were expressed as mean ± standard deviation; non-normality data were expressed as median and quartiles. Differences in categorical variables (e.g., postoperative complications) were assessed by Wilcoxon rank-sum test or Fisher exact test, whereas differences in continuous variables were assessed by Student’s t-test when the assumption of normality was valid. p-values < 0.05 were considered significant.

## Results

### General information

10 males and 6 females with a mean age of 39.4 years (19–62 years) were treated with M-INFIX; 14 males and 6 females with a mean age of 38.4 years (18–68 years) were treated with C-INFIX. According to AO/OTA typing, in the M-INFIX group, there were 9 cases of type 61B (5 cases of type B1 and 4 cases of type B2) and 7 cases of type 61C1; in the C-INFIX group, there were 10 cases of type 61B1 (7 cases of type B1 and 3 cases of type B2) and 10 cases of type 61C1. The mean follow-up time was 15.2 ± 2.6 months and 15.4 ± 3.7 months in the M-INFIX and C-INFIX groups, respectively; the mean ISS scores were 11.6 ± 3.5 and 10.8 ± 3.4, respectively. The mean fracture healing time was 3.9 ± 1.0 and 3.8 ± 1.1 months, respectively. There was no statistical significance (p > 0.05) in gender, age, fracture typing, follow-up time, ISS score, and fracture healing time between the two groups. The operative time was 47.5 ± 8.5 min and 63.6 ± 8.5 min, and intraoperative blood loss was 18.1 ± 3.3 min and 40.4 ± 9.0 min in the M-INFIX and C-INFIX groups, respectively. The operative time and intraoperative blood loss were statistically significant between the two groups (p < 0.001) (Table 1).

**Table 1:** Patient General Information

Parameter	C-INFIX (n = 20)	M-INFIX (n = 16)	*t/χ*^*2*^ value	*p* value
Gender(n,%)			2.363	0.159^a^
male	4(20.0)	7(43.7)		
female	16(80.0)	9(56.3)		
Age (years)	43.4 ± 15.8	42.9 ± 18.7	0.080	0.937^b^
OTA/AO classification (n, %)			1.406	0.236^c^
61B (B1/B2)	7 (35.0)/ 3 (15.0)	5(31.2)/4 (25.0)		
61C1	10(50.0)	7(43.8)		
ISS	10.8 ± 3.4	11.6 ± 3.5	−0.675	0.504^b^
Operation time (min)	47.5 ± 8.5	63.6 ± 8.5	−5.082	0.001^b^
Intraoperative blood loss (ml)	18.1 ± 3.3	40.4 ± 9.0	−9.335	0.001^b^
Fracture healing time(month)	3.9 ± 1.0	3.8 ± 1.1	0.276	0.784^b^
Follow-up time(month)	15.4 ± 3.7	15.2 ± 2.6	0.148	0.883^b^

^a^Fisher’s exact test

^b^Independent-Sample T Test

^c^Pearson chi-squared test

### Complications of surgery

In the M-INFIX group, there were 2 (12.5%) cases of surgical complications, including 1 skin infection at the site of the pubic symphysis wound, which was considered to be a superficial skin infection and was treated as an outpatient with simple debridement and disappeared after oral antibiotics; 1 case of LFCN, in which the patient developed abnormal skin sensation in the anterolateral thigh, which disappeared after removal of the internal fixation. A total of 10 (50.0%) surgical complications occurred in the C-INFIX group, including 2 cases of skin infection with mild redness and oozing at the wound, which disappeared after active dressing changes and oral antibiotics. 6 patients had anterolateral femoral cutaneous nerve injury (including 2 patients with bilateral lateral femoral cutaneous nerve injury), 5 patients had symptoms that disappeared after removal of the internal fixation, and 1 patient complained of mild skin sensation and numbness after removal of the internal fixation. One patient presented with symptoms of femoral nerve palsy, mainly in the form of decreased muscle strength of the quadriceps, and a electromyography showed damage to the femoral nerve. The possible causes were insufficient curvature of the titanium rods during molding, which resulted in the close proximity of the femoral nerve to the titanium rods, as well as untimely management of abdominal distention after surgery, which led to irritation of the femoral nerve. After removal of the internal fixation, the muscle strength returned to normal. One patient had deep vein thrombosis in the lower extremity, which was treated with symptomatic treatment such as placement of an inferior vena cava filter and anticoagulation, and the vascular patency and thrombosis disappeared after 3 months of postoperative review. The difference between M-INFIX and C-INFIX in terms of complication rate was statistically significant (p = 0.032) (Table 2).

**Table 2:** Incidence of complications

Complication	C-INFIX (n = 20)	M-INFIX (n = 16)	*χ* ^*2*^	*P* value
Total	10	2	5.625	0.032^a^
LFCN injury	6	1		
Wound infection	2	1		
Deep vein thrombosis	1	0		
Heterotopic ossification	1	0		

^a^Fisher’s exact test

### Quality of restoration and functional score

The quality of repositioning was mainly evaluated according to the Matta score on postoperative X-ray and CT after removal of the internal fixation. 6 cases in the M-INFIX group were excellent, 6 cases were good, 3 cases were acceptable, and 1 case was poor, with an average displacement distance of 6.1(3.3,10.2) mm, which was an excellent rate of 75%. 1 case in the C-INFIX group was excellent, 7 cases were good, 7 cases were acceptable, and 5 cases were poor, with an average displacement distance of 11.8(6.6, 19.7) mm, which was an excellent rate of 40%. The differences in average displacement distance and excellent rate between the two groups were statistically significant (p < 0.05). (Table 3).

**Table 3:** Quality of postoperative fracture reduction

Group	Total	Matta score						
		Excellent	Good	Acceptable	Poor	Average displacement distance [*M(P25,P75)*, mm]		Outstanding rate(%)
C-INFIX	20	1	7	7	5	11.8(6.6,19.7)	40	
M-INFIX	16	6	6	3	1	6.1(3.3,10.2)	75	
*χ* ^*2*^ */Z*	−2.589					−2.675	4.410	
*P* value	0.010^d^					0.007^d^	0.036^c^	

^c^Pearson chi-squared test

^d^Wilcoxon rank-sum test

Functional scores were assessed according to the patients at the final follow-up. The mean Majeed score in the M-INFIX group was 80.5 (66.8, 90.8), with an excellent rate of 75%; the mean Majeed score in the C-INFIX group was 87.0 (76.5, 91.8), with an excellent rate of 80%, and there was no statistical significance in the difference between the two groups (p > 0.05). (Table 4).

**Table 4:** Functional Assessment

Group	Total	Majeed score					
		Excellent	Good	Acceptable	Poor	Average score *[M(P25,P75)]*	Outstanding rate(%)
C-INFIX	20	12	4	3	1	87.0 (76.5, 91.8)	80
M-INFIX	16	7	5	2	2	80.5 (66.8, 90.8)	75
*χ* ^*2*^ */Z*		−0.906				−0.813	0.129
*P* value		0.365^d^				0.416^d^	1.000

^d^Wilcoxon rank-sum test

### The condition of the internal fixation

No loosening or failure of the internal fixation was observed during the treatment period in either the M-INFIX or C-INFIX groups. The mean time to removal of the internal fixation was 6.2 ± 2.8 months in the M-INFIX group and 6.1 ± 2.7 months in the C-INFIX group. All patients were followed up completely.

## Disscusion

INFIX has been gradually adopted by clinicians for its advantages of reduced soft tissue injury, less bleeding, lower incidence of medically induced vascular injury, ease of operation, and ease of care [20, 21]. After more than 10 years of clinical application, it has been proven to be a rapid and convenient way to stabilize the anterior pelvic ring with good biomechanical stability and clinical outcomes, and is now an alternative option for anterior pelvic ring fractures [14, 22].

Current indications for INFIX include emergency management of hemodynamically unstable pelvic ring ruptures and combined fixation of vertical and rotational instability of the pelvis combined with anterior ring injuries [23]. In the obese patient population, INFIX is more acceptable because it is placed subcutaneously, effectively avoiding the inconvenience of external fixation [24]. Despite its advantages, complications of INFIX surgery have been reported to be not uncommon [25]. Among them, LFCN injury is the most common complication of INFIX, and Vaidya noticed 27 cases of LFCN injury with an incidence of 29.7% through postoperative analysis of 91 patients. Christian et al. reported LFCN injury in 14 out of 29 patients with an incidence of 48.3%. To reduce the incidence of LFCN injury, Apivatthakakul et al. performed simulated INFIX placement through 15 fresh cadaveric specimens and measured the distance of rods and rods from important neurovascular vessels. It was found that the depth of the pedicle screw is closely related to the degree of nerve compression, and it is more difficult to perform rod-rod connection with a single-axis pedicle screw, so he recommended the use of multi-axis pedicle screws and the screws should be at a certain distance from the bone surface [16]. Osterhoff et al. found that the effect on muscle tissue and neurovascular bundles was minimal at a rod-bone distance of 2 cm by studying different rod-bone distances, and also pointed out that LFCN is sometimes compressed by the rod protruding from the lateral side of the screw head, so it is recommended that the rod on the lateral side of the nail-rod connection should be shorter [26]. Our study found that the M-INFIX group had a lower incidence of LFCN injury than the C-INFIX group (6.3% vs. 30.0%). The lower incidence of LFCN injury in the M-INFIX group may be due to the fact that the distance between the screw and the bone surface was kept at about 2 cm, which reduced the chance of LFCN irritation by the tail of the nail; and the smaller curvature allowed for easier placement of the nail-rod connection, avoiding more soft tissue stripping [27]. The smaller curvature also made it easier to place the rod connection, avoiding more soft tissue stripping; the rod tail and LFCN alignment were basically the same, reducing the possibility of LFCN irritation due to limb activity at a later stage [26].

The incidence of other surgical complications was low, with reports showing a femoral nerve palsy incidence of approximately 1.6%, heterotopic ossification of 24.7%, and infection of 3% [14]. Skin infections occurred in both the C-INFIX and M-INFIX groups, but were superficial subcutaneous infections that healed well with simple debridement of the wound. One case of femoral nerve palsy occurred in the C-INFIX group, and the patient recovered after removal of the internal appliance. In the Hess study, a series of six patients with femoral nerve palsy were described, and only half recovered despite removal of the insertion. Femoral nerve palsy is an uncommon clinical complication, and the reasons for its occurrence are not fully understood. In most cases, the surgical dissection during percutaneous placement of internal fixation will be limited to the subcutaneous level, and deeper dissections are often inaccessible, leading to the possibility of femoral nerve entrapment. Screws placed at a distance of 2 cm from the bone surface, rods placed in the “bikini” area, and timely postoperative management of abdominal distension will reduce the incidence of femoral nerve palsy. The absence of ectopic ossification in our study may be related to the routine use of postoperative NSAIDs for prophylaxis and careful intraoperative handling [28].

Despite the large number of anatomical and clinical studies on INFIX, there are no good measures to reduce the incidence of complications on the neurological side in the surgical operation of C-INFIX [29, 30]. Moreover, the long span of the rod may lead to difficulties in ensuring the quality of fracture repositioning [31]. Therefore, we proposed the surgical approach of M-INFIX, in which the screws are changed from the contralateral iliac bone to both sides of the pubic symphysis, which has the advantage of making the rods shorter and easier to shape and reset the fracture, while obtaining better biomechanical stability. Christopher found, after comparing different INFIX approaches to fixing anterior pelvic ring fractures, that M-INFIX was more effective than C- INFIX had better rotational stability and less mean displacement under the same load than C-INFIX, confirming that M-INFIX has better biomechanical stability than C-INFIX [20]. In our study, we also found that the M-INFIX group had higher fracture reduction scores and lower surgical complication rates than the C-INFIX group.

The study still has some limitations: (1) the surgical indications are narrow, M-INFIX is suitable for unilateral anterior pelvic ring injury, and C-INFIX fixation is still recommended for bilateral anterior pelvic ring injury; (2) the dissection at the pubic symphysis is an additional injury with the possibility of damaging the bladder and other organs; (3) surgical removal of the internal fixation is still required; (4) the sample size is small, and a multicenter, large sample is still needed studies.

## Conclusions

In conclusion, this study demonstrated positive clinical outcomes such as higher quality of fracture reduction and lower incidence of surgical complications, as well as higher biomechanical stability with M-INFIX compared to C-INFIX. These findings indicate that M-INFIX is an alternative option for the treatment of unilateral anterior ring instability injuries of the pelvis and warrants further investigation.

## Data Availability

The datasets used and/or analysed during the current study are available from the corresponding author on reasonable request.

## Declarations

### Statements

All methods were performed in accordance with the relevant guidelines and regulations.

## Abbreviations

M-INFIX: Modified anterior subcutaneous internal fixation
C-INFIX: Conventional anterior subcutaneous internal fixation
LFCN: Lateral femoral cutaneous nerve palsy
CT: Computed tomography
ISS: Injury Severity Score
